# *OsRNE* Encodes an RNase E/G-Type Endoribonuclease Required for Chloroplast Development and Seedling Growth in Rice

**DOI:** 10.3390/ijms26052375

**Published:** 2025-03-06

**Authors:** Huimin Fang, Lili Song, Kangwei Liu, Yishu Gu, Yao Guo, Chao Zhang, Long Zhang

**Affiliations:** 1 Guangling College, Yangzhou University, Yangzhou 225000, China; hmfang@yzu.edu.cn; 2 Key Laboratory of Crop Genetics and Physiology of Jiangsu Province, Key Laboratory of Plant Functional Genomics of the Ministry of Education, Yangzhou University, Yangzhou 225009, China; 18300765057@163.com (L.S.); lkw18352768872@163.com (K.L.); yi09402024@163.com (Y.G.); 15554430252@163.com (Y.G.); 3 Co-Innovation Center for Modern Production Technology of Grain Crops of Jiangsu Province, Joint International Research Laboratory of Agriculture & Agri-Product Safety of the Ministry of Education, Yangzhou University, Yangzhou 225009, China

**Keywords:** chlorophyll biosynthesis, chloroplast development, rice, RNA metabolism, RNase E/G, thylakoid

## Abstract

Chloroplast biogenesis is a crucial biological process in plants. Endoribonuclease E (RNase E) functions in the RNA metabolism of chloroplast and plays a vital role for chloroplast development in *Arabidopsis*. However, despite sharing 44.7% of its amino acid sequence identity with *Arabidopsis* RNase E, the biological function of rice OsRNE (*Oryza sativa* RNase E) remains unknown. Here, we identified a *white leaf and lethal 1* (*wll1*) mutant that displayed white leaves and died at the seedling stage. The causal gene *OsRNE* was isolated by MutMap+ method. CRISPR/Cas9-mediated knockout of *OsRNE* resulted in white leaves and seedling lethality, confirming *OsRNE* as the causal gene for the *wll1* phenotype. The albino phenotype of *osrne* mutant was associated with decreased chlorophyll content and abnormal thylakoid morphology in the chloroplast. The absence of *OsRNE* led to a significant reduction in the Rubisco large subunit (RbcL), and the 23S and 16S chloroplast rRNAs were nearly undetectable in the *osrne* mutant. *OsRNE* transcripts were highly expressed in green tissues, and the protein was localized to chloroplasts, indicating its essential role in photosynthetic organs. Furthermore, transcriptome analysis showed that most of the genes associated with photosynthesis and carbohydrate metabolism pathways in the *osrne* mutant were significantly down-regulated compared with those in WT. Chlorophyll- and other pigment-related genes were also differentially expressed in the *osrne* mutant. Our findings demonstrated that OsRNE plays an important role in chloroplast development and chlorophyll biosynthesis in rice.

## 1. Introduction

Chloroplasts are essential for photosynthesis in higher plants, converting light energy into chemical energy [1]. Chlorophyll, synthesized within chloroplasts, determines leaf color and influences seedling growth. Any deficiency in chloroplast development leads to leaf color mutants, and many mutants have been identified, e.g., pale green, yellowing, white-striped, variegated, and albino mutants [2,3,4,5,6]. All of these mutants, especially albino mutants, are ideal specimens for studying potential molecular mechanisms of chloroplast and leaf development [7,8,9,10,11].

Chloroplast development occurs in three stages: (1) plastid DNA replication and synthesis, (2) chloroplast assembly, and (3) photosynthetic apparatus formation [12,13]. In the second stage, nuclear-encoded RNA polymerase (NEP) preferentially transcribes plastid housekeeping genes to promote transcription and translation in chloroplasts. In the third stage, plastid-encoded RNA polymerase (PEP) combines with the nuclear encoded protein to form a photosynthetic and metabolic system that controls chloroplast development. Chloroplasts are semi-autonomous organelles and have independent small genomes, but most of the proteins are encoded in the nucleus and transferred to chloroplasts after translation. Taking rice as an example, the chloroplast genome is about 135 kb, containing 34 RNA-coding genes and 120 protein-coding genes [14]. Approximately 3000 proteins play an important role in chloroplast functions, of which over 95% are encoded by nuclear genes [15]. In summary, chloroplast development is coordinately regulated by both plastid and nuclear-encoded genes. Therefore, cloning and identifying these nuclear genes will help elucidate the complex regulatory mechanisms of plant chloroplast development.

Post-transcriptional regulation plays a major role in chloroplast biogenesis, with nucleus-encoded ribonucleases facilitating RNA processing and degradation [16]. Endonucleases are divided into CSP41, ribonuclease E/G (RNase E/G), RNase III, RNase H, RNase J, RNase P, and RNase Z, and they have various functions in RNA metabolism, including maturation of rRNA and tRNA precursors, processing of polycistronic mRNAs, and RNA decay [16]. RNase E/G proteins contain RNase E and its truncated form, RNase G, and have been reported to play an important role in RNA metabolism in bacteria, cyanobacteria, and *Arabidopsis* [17,18,19,20]. In *Escherichia coli*, RNase E encoded by the *RNE* gene is a ribosomal RNA (rRNA)-processing enzyme. RNase E has RNA-binding activity, cleaves RNA endoribonucleolytically in AU-rich single-stranded regions, and interacts with polynucleotide phosphorylase (PNPase) and other proteins implicated in RNA processing and degradation [21,22,23,24,25,26,27]. *Anabaena* 3′–5′ exoribonuclease RNase II binds to RNase E and modulates its endoribonucleolytic activity [18]. *Arabidopsis* RNase E is located in the chloroplast as a high-molecular-weight complex [19]. Absence of RNase E results in multiple defects in chloroplast RNA metabolism in *Arabidopsis* [19,20,28]. Most importantly, polycistronic precursor transcripts over-accumulate in the knockout plants of *Arabidopsis*, while several mature monocistronic mRNAs are strongly reduced [20]. The mutant also exhibits defective transcript processing of mRNAs for ribosomal proteins, which results in plastid ribosome deficiency and impaired plant growth [20,28]. There is only a single RNase E/G gene in the nuclear genomes of higher plants and not in eukaryotes lacking chloroplasts, which implies that RNase E may play an important role in the chloroplast development of higher plants. However, little is known about the role of RNase E in rice.

In this study, we cloned the *OsRNE* gene through MutMap+ method, confirmed the function of *OsRNE* by CRISPR/Cas9 gene editing approach, and conducted a detailed study of seedling phenotypes. Meanwhile, a functional analysis of OsRNE, including expression pattern and subcellular localization, was carried out. Finally, RNA-seq was used to analyze the expression levels of genes related to chlorophyll and other pigments as well as genes involved in photosynthesis and carbohydrate metabolism pathways in *osrne* mutants. Our findings demonstrate that OsRNE plays an important role in chloroplast development, chlorophyll synthesis, and photosynthesis in rice.

## 2. Results

### 2.1. Genetic Mapping of the White Leaf Locus Using the MutMap+ Method

**Figure 1:**
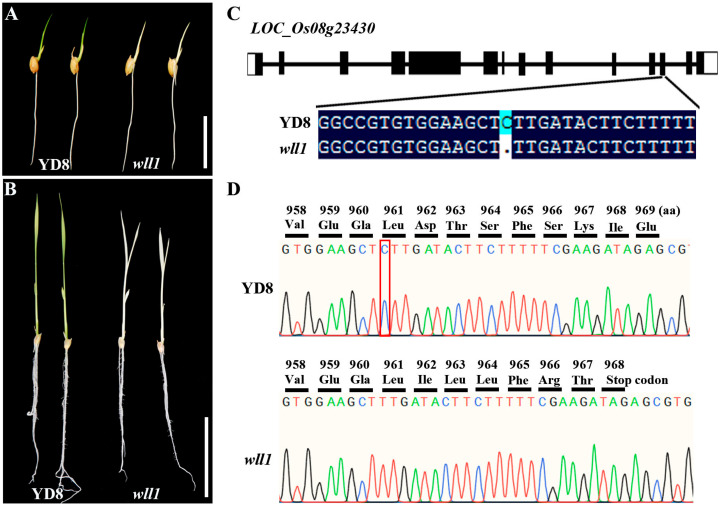
Isolation of causal mutation in white leaf phenotype through MutMap+ method. (**A**,**B**) Comparison of the four-day-old (**A**) and ten-day-old (**B**) seedlings of YD8 and *wll1*. Scale bars = 2 cm (**A**) and 5 cm (**B**). (**C**) Gene structure of *LOC_Os08g23430*. The candidate Indel (a single base C deletion) locates at the 12th exon of *LOC_Os08g23430* gene. (**D**) Sequence chromatograms of *wll1* mutation. The transcript of the mutant would encode a protein with 967 amino acids. Red box represents the missing single base C.

The *wll1* was isolated from a ^60^Co-irradiated mutant pool of japonica variety Yandao 8 (YD8). Under paddy conditions, the seedlings of *wll1* exhibited an obvious white leaf phenotype from the emergence of the first leaf compared with the green leaves of YD8 plants and died within three weeks (Figure 1A,B). Because *wll1* is a lethal mutant, heterozygous plants were used to generate progeny for genetic analysis and mapping. The segregation of green and white phenotypes (73 green: 19 white; X^2^ = 0.93 < X^2^_0.05,1_ = 3.84) confirmed that the mutation is controlled by a recessive nuclear gene. MutMap+, a method suitable for identifying mutations that cause early development lethality or generally hamper crossing, was adopted to identify the causal gene [29]. The genomic DNA of 30 green leaf progenies and 30 white leaf progenies of the heterozygous plants were mixed in equal proportion to produce DNA pool 1 and pool 2, respectively. The DNA samples of pool 1 and pool 2 were separately sequenced to generate Illumina HiSeq paired-end reads that were mapped to reference genomes of Nipponbare (http://rice.uga.edu (accessed on 3 December 2023)). The numbers of clean reads obtained from DNA pool 1 (clean base 12.14 G) and pool 2 (clean base 16.03 G) were 80,915,726 and 106,896,834, respectively. The sequence depth of two DNA pools was 33 x and 43 x, respectively, corresponding to the rice genome (approximately 370 Mb). SNP/Indel index was calculated as the ratio between the number of reads of a mutant SNP/Indel and the total number of reads corresponding to the SNP/Indel. Drawing upon the fundamental principles of genetics, we established specific criteria to pinpoint the causal mutation underlying the *wll1* phenotype (Supplementary Figure S1A). These criteria are outlined as follows: (1) an SNP/Indel-index of 1 in the mutant pool, as the phenotype of albino seeding is homozygous mutation (*wll1/wll1*), and (2) an SNP/Indel-index of less than 0.5 in the wild-type pool, accounting for the presence of homozygous wild-type sequences (*WLL1/WLL1* with SNP/Indel-index = 0) and heterozygotes (*WLL1/wll1* with SNP/Indel-index of 0.5). After this filtration, only one Indel, with Indel-index = 1 (23 mutation reads/23 total reads) and 0.30 (3 mutation reads/10 total reads) in the mutant pool and wild-type pool, respectively, was identified (Supplementary Figure S1B). This Indel represents a single base G deletion in 14,169,090th bp of chromosome 8.

To verify the results of MutMap+, we randomly selected 10 *wll1* seedlings and amplified the Indel using a specific pair of primers for Sanger sequencing. All mutant seedings contained the homozygous mutation of the candidate Indel, suggesting that the mutant phenotype co-segregated with the genotype.

Based on the rice genome annotation project (https://rapdb.dna.affrc.go.jp, accessed on 1 January 2024), the candidate Indel, 14,169,090th bp of chromosome 8, is located at the 12th exon of *LOC_Os08g23430* gene (Figure 1C,D). The rice genome database annotates that *LOC_Os08g23430* encodes the rice OsRNE protein, belonging to the RNase E/G endonuclease family. The *OsRNE* gene comprises 14 exons and 13 introns (Figure 1C). The full length of its CDS is 3258 bp and encodes a protein of 1085 amino acids. Mutation of the candidate Indel in *wll1* resulted in frame shifts with premature termination (Figure 1C). If translated, the transcript in the mutant would encode a protein with 967 amino acids (Figure 1D).

### 2.2. Functional Validation of OsRNE Gene Using CRISPR-Cas9 System

**Figure 2:**
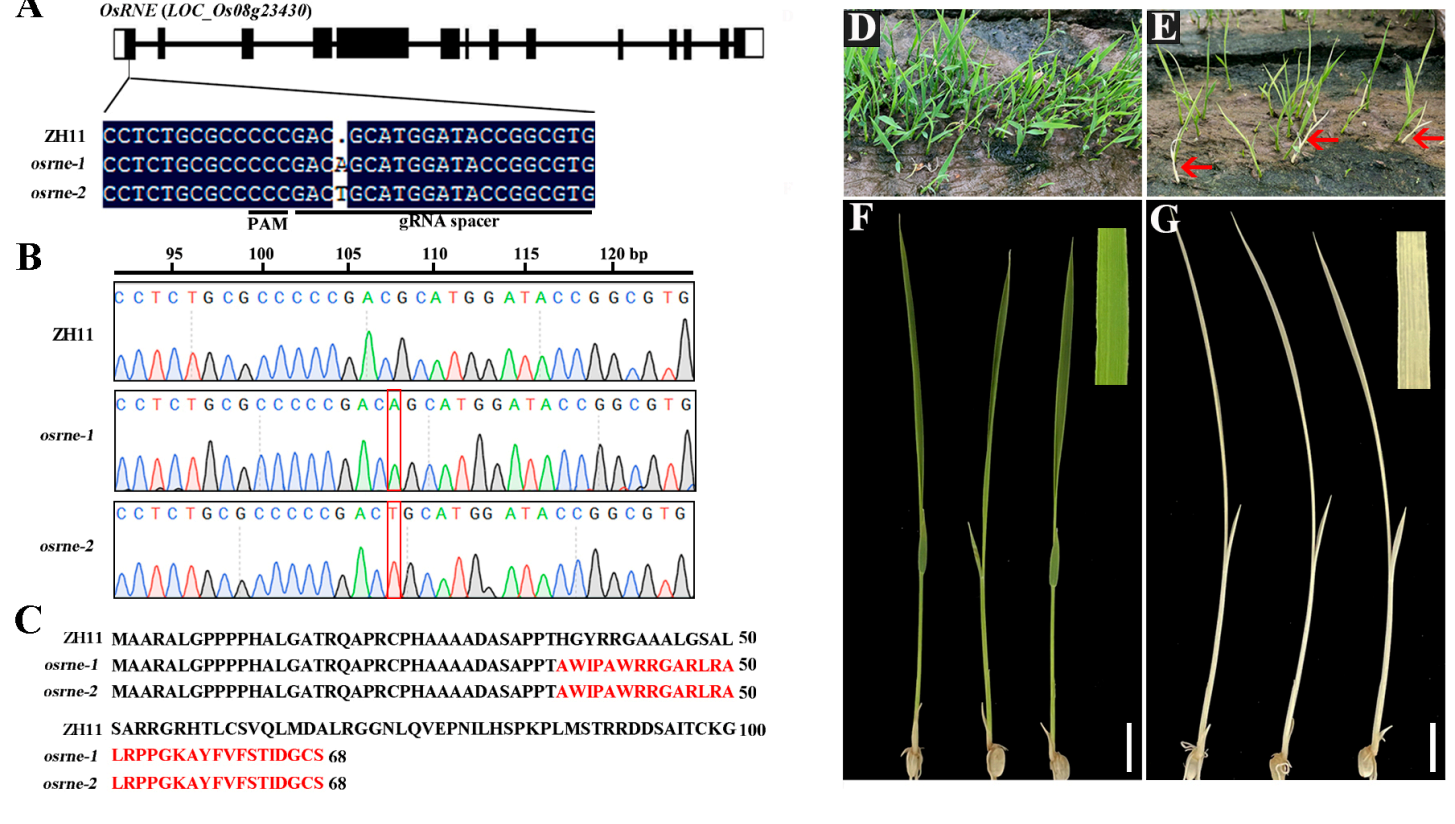
Knockout of *OsRNE* leads to albino phenotype. (**A**) Two independent lines were generated by CRISPR/Cas9 in the first exon of the *OsRNE* gene. (**B**) Mutation events were confirmed by Sanger sequencing. Red box represents the insertion of single base. (**C**) The transcript of *osrne* mutants would encode a protein with 68 amino acids. Red fonts show the amino acids formed by the frameshift mutation. The full-length CDS of *OsRNE* encodes a protein of 1085 amino acids, and Figure 2C shows the first 100 amino acids in ZH11. (**D**–**G**) Phenotypic analyses of WT and *osrne-1* seedlings. (**D**,**E**) Seedlings of WT (**D**) and heterozygous mutant (**E**) at the two-leaf stage in the field. The red arrows represent white seedlings separated from the heterozygous mutant. (**F**,**G**) The leaves of normal green (**F**) and white seedlings (**G**) at the two-leaf stage. Scale bars = 10 mm (**F**,**G**).

To further investigate the biological function of OsRNE in rice, a clustered, regularly interspaced, short palindromic repeats (CRISPR)/CRISPR-associated protein 9 (Cas9)-mediated gene editing system was used to generate osrne mutants. The guide RNA (gRNA) expression cassettes that specifically target the first exon of OsRNE were generated and transformed into rice variety Zhonghua 11 (wild-type, WT) (Figure 2A). Two independent homozygous mutant lines *osrne-1* and *osrne-2*, derived from the self-progeny population of heterozygous plants, were identified with an adenosine (A) and thymine (T) insertion in the 108th bp of the first extron, respectively (Figure 2B), which both resulted in frame-shifts with premature termination (Figure 2C). If translated, this transcript would encode a protein with 68 amino acids and does not include most of the domains in OsRNE (Figure 2C). As the two homozygous mutants caused the same type of premature termination, the mutant line *osrne-1* was used for further analysis.

The seedlings of WT grew normally, while the heterozygous mutant seedlings showed phenotypic separation of green and white (Figure 2D,E). Sanger sequencing showed that the *OsRNE* gene in the white seedlings underwent homozygous mutations, whereas normal green seedlings were either homozygote or heterozygote for the *OsRNE* allele. All the green seedlings grew normally to maturity, similar to the recipient parent Zhonghua 11, while the white seedlings did not survive past the three-leaf stage (Figure 2F,G). The knockout lines exhibited the same seedling phenotype as the *wll1* mutant, further demonstrating that *LOC_Os08g23430* is the gene responsible for the *wll1* phenotype.

### 2.3. Phenotypic Characterization of Osrne Mutant

**Figure 3:**
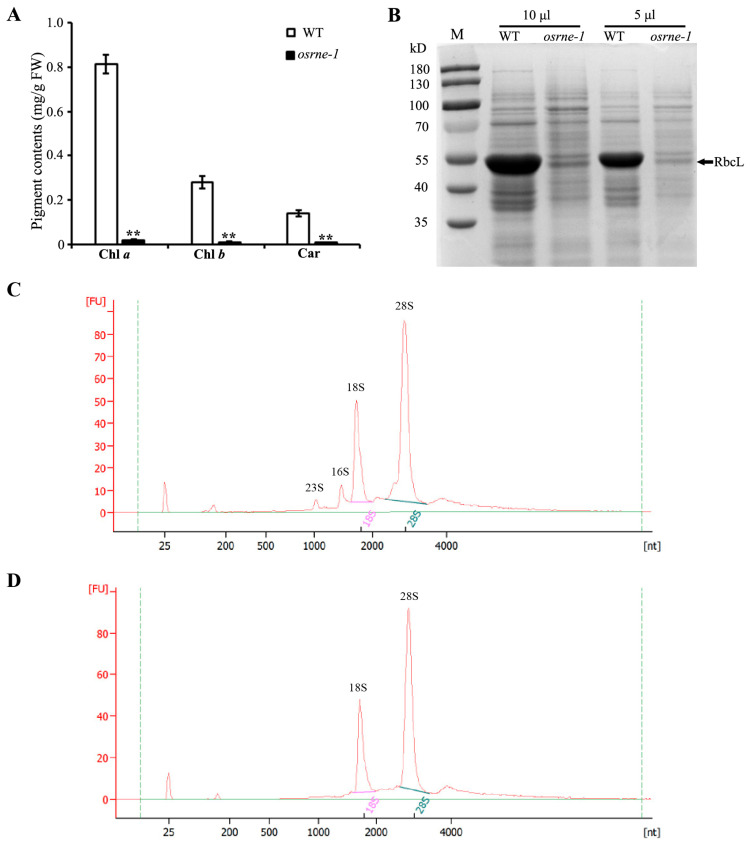
Analyses of pigment contents and rRNA from WT and *osrne-1* mutant. (**A**) Pigment contents in leaves at the two-leaf stage. Chl a, chlorophyll a; Chl b, chlorophyll b; Car, carotenoid. Error bars represent SD (standard deviation) of three biological replicates. Asterisks indicate a significant difference between the WT and *osrne-1* by Student’s *t*-test; ** *p* < 0.01. (**B**) SDS-PAGE analysis of total proteins in leaves WT and *osrne-1* mutant. (**C**,**D**) rRNA analysis using Agilent 2100 in WT (**C**) and *osrne-1* (**D**) leaves at the two-leaf stage.

To assess the impact of the *OsRNE* mutation on pigment biosynthesis, we measured chlorophyll and carotenoid levels in WT and *osrne-1* leaves at the two-leaf stage. The levels of chlorophyll a (Chl a), chlorophyll b (Chl b), and carotenoids (Car) were significantly lower in *osrne-1* than in WT (Figure 3A), indicating a critical role for OsRNE in chlorophyll biosynthesis. Moreover, limited Rubisco large subunit RbcL proteins were found in the *osrne-1* leaves at the two-leaf stage (Figure 3B), which revealed that OsRNE might also play an important role in regulating Rubisco formation.

Eukaryotic ribosomes consist of a 60S large subunit and a 40S small subunit. The 60S and 40S subunits comprise rRNAs (28S, 5.8S, 5S, and 18S) and ribosomal proteins, while chloroplast ribosomes consisting of a 50S large subunit and a 30S small subunit comprise rRNAs (23S, 5S, and 16S) and ribosomal proteins. We then analyzed the composition and content of rRNAs using an Agilent 2100 (Agilent Technologies, Santa Clara, CA, USA) in WT and *osrne-1* leaves at the two-leaf seedling stage. The 28S and 18S rRNAs were almost the same in WT and *osrne-1* leaves (Figure 3C,D). However, the chloroplast rRNAs (23S and 16S) could not be detected in *osrne-1* leaves (Figure 3D). These results indicate that *osrne-1* was defective in the biogenesis of chloroplast ribosomes.

### 2.4. Chloroplast Development Was Impaired in Osrne Mutant

**Figure 4:**
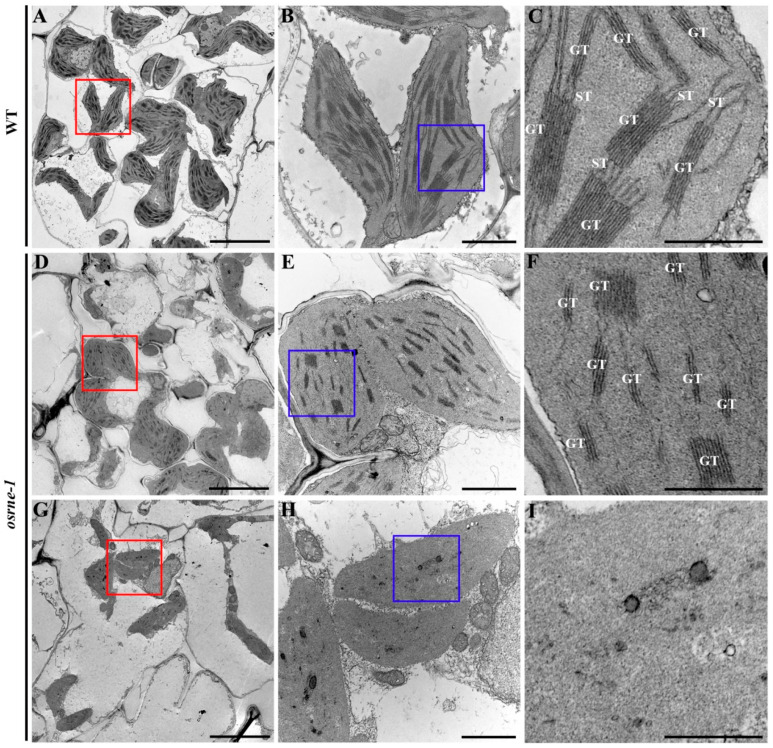
Chloroplasts ultrastructure in WT and *osrne-1* mesophyll cells at the two-leaf stage. (**B**,**E**,**H**) represent the magnified regions indicated by red outline in (**A**,**C**,**E**), respectively. (**C**,**F**,**I**) represent the magnified regions indicated by blue outline in (**B**,**E**,**H**), respectively. GT, grana thylakoid; ST, stroma thylakoid. Scale bars = 5 μm (**A**,**D**,**G**), 1 μm (**B**,**E**,**H**) and 0.5 μm (**C**,**F**,**I**).

As a consequence of the deficiency in chlorophyll biogenesis in the mutant, we further investigated the ultrastructure of the chloroplast using transmission electron microscopy (TEM). WT chloroplasts exhibited well-developed grana thylakoids and stroma thylakoids (Figure 4A–C), whereas *osrne-1* mutants displayed two distinct phenotypes: one with short grana thylakoids and almost unobservable stroma thylakoids (Figure 4D–F) and another with a nearly complete absence of thylakoids (Figure 4G–I). These results imply that OsRNE is essential for chloroplast development from the beginning of seedling establishment.

### 2.5. Expression Analysis and Subcellular Localization of OsRNE

**Figure 5:**
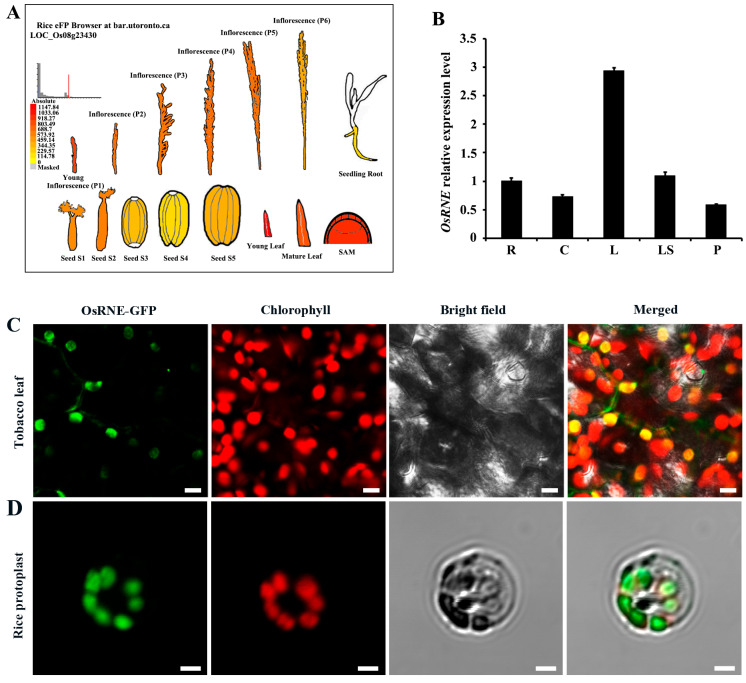
Expression pattern of *OsRNE* and subcellular localization of OsRNE. (**A**) Expression patterns of *OsRNE* based on the rice eFP browser. (**B**) qRT-PCR analysis of *OsRNE* relative expression level in different tissues at the heading stage. R, roots; C, culms; L, flag leaves; LS, leaf sheaths; P, panicles before heading. Error bars represent ± SD (*n* = 3). Confocal microscope images showing the subcellular localization of OsRNE-GFP in tobacco leaf (**C**) and rice protoplast (**D**). Signals from GFP fluorescence, chlorophyll autofluorescence, bright field, and merged images are shown. Scale bars = 10 µm (**C**) and 5 µm (**D**).

Predicting through the Rice eFP Browser (http://bar.utoronto.ca/efprice/, accessed on 6 March 2024), the transcripts of *OsRNE* were highly expressed in young leaf, young inflorescence, mature leaf, and shoot apical meristem (SAM) (Figure 5A). To confirm the expression pattern of *OsRNE*, total RNA was extracted from different organs of WT, including the roots, culms, leaves, leaf sheaths, and panicles at the heading stage. Quantitative RT-PCR (qRT-PCR) analysis showed that *OsRNE* exhibited constitutive expression (Figure 5B). The highest expression was observed in the leaves, while relatively lower expression was detected in the culms, panicles, and roots (Figure 5B). These data suggest that OsRNE plays an important role in photosynthetic organs.

The OsRNE contains a carbohydrate-binding module 2 (CBM2) domain at the N-terminal and a RNaseE/G domain at the C-terminal (Supplementary Figure S2). It has been reported that proteins containing CBM domains are localized in chloroplasts [30,31]. According to the prediction in WoLF PSORT (https://wolfpsort.hgc.jp/, accessed on 12 May 2024), the OsRNE is a chloroplast localization protein (Supplementary Figure S3). To explore the subcellular localization of the OsRNE protein, the N-terminal 1-206 amino acids of OsRNE containing a chloroplast transit peptide (cTP) was fused to the green fluorescent protein (GFP) and transformed into *N*. *benthamiana* leaves and rice protoplasts. Fluorescent signals of the OsRNE-GFP fusion protein merged with chloroplast autofluorescence (Figure 5C,D), indicating that the OsRNE protein is a chloroplast-targeted protein.

### 2.6. The Mutation of OsRNE Altered the Expression of Photosynthesis and Carbohydrate Metabolism Pathway Genes

**Figure 6:**
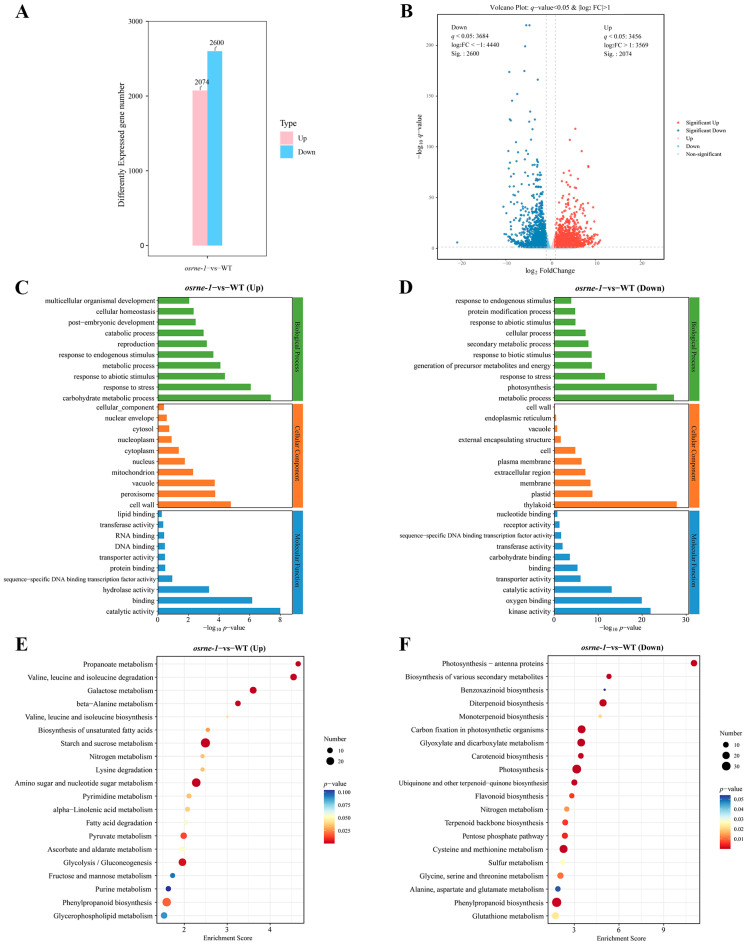
Transcriptome analysis of WT and *osrne-1* seedlings. (**A**) Number of DEGs from comparison of *osrne-1* and WT seedling transcriptomes. (**B**) Volcano plot of significantly up- or down-regulated DEGs. (**C**) GO enrichment analysis of the up-regulated DEGs. (**D**) GO enrichment analysis of the down-regulated DEGs. (**E**) KEGG pathway enrichment analysis of the up-regulated DEGs. (**F**) KEGG pathway enrichment analysis of the down-regulated DEGs.

To analyze the effect of the *OsRNE* mutation on gene expression, we compared the transcriptional profiles of nuclear and plastidic genes in *osrne-1* and WT seedlings using RNA-seq. A total of 4674 differentially expressed genes (DEGs, *q* < 0.05; |log_2_(FoldChange)| ≥ 1) were identified between *osrne-1* and WT. Among the 4674 DEGs, 2074 and 2600 genes were up-regulated and down-regulated, respectively (Figure 6A, Supplementary Tables S1 and S2). The volcano plot shows distribution of a statistical significance and the altered expression level of the DEGs between *osrne-1* and WT (Figure 6B).

Gene ontology (GO) analysis of the 4674 DEGs identified carbohydrate metabolic process (96 DEGs) for biological processes, cell wall (84 DEGs) for cellular components, and catalytic activity (282 DEGs) for molecular function as the most significant up-regulated DEGs (Figure 6C) in *osrne-1* vs. WT. Among the down-regulated DEGs, metabolic process (842 DEGs) and photosynthesis (73 DEGs) for biological processes, thylakoid (132 DEGs) for cellular components, and kinase activity (295 DEGs) for molecular function were the most significant (Figure 6D). In the significantly enriched GO, terms of down-regulated DEGs, metabolic process, photosynthesis, thylakoid, plastid, kinase activity, and oxygen binding, which are associated with photosynthesis and carbon fixation, were obviously identified (Figure 6D).

For KEGG pathway analysis, the top 20 significant categories were identified for up- and down-regulated DEGs. The up-regulated DEGs included propanoate metabolism, valine and leucine and isoleucine degradation, galactose metabolism, starch and sucrose metabolism, amino sugar and nucleotide sugar metabolism, and glycolysis/gluconeogenesis (Figure 6E). On the contrary, photosynthesis-antenna proteins, diterpenoid biosynthesis, photosynthesis, carbon fixation in photosynthetic organisms, biosynthesis of various secondary metabolites, carotenoid biosynthesis, ubiquinone and other terpenoid-quinone biosynthesis, and flavonoid biosynthesis were identified in the down-regulated DEGs (Figure 6F).

**Figure 7:**
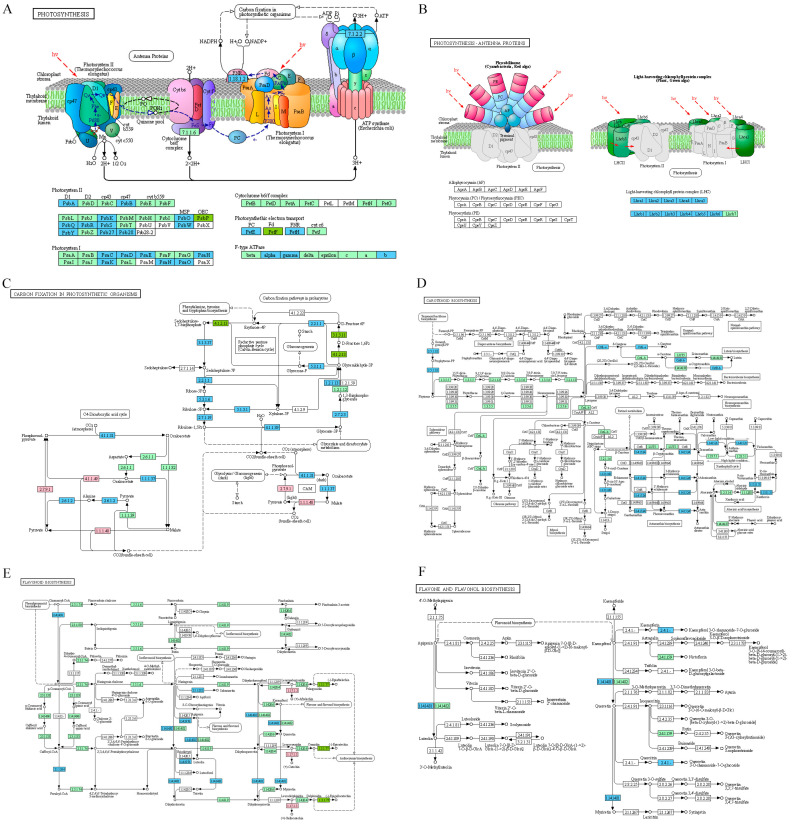
Differential map of genes in photosynthesis and carbohydrate metabolism pathways between WT and *osrne-1*. (**A**) Differential genes in photosynthesis pathway. (**B**) Differential genes of photosynthesis-antenna proteins. (**C**) Differential genes of carbon fixation in photosynthetic organisms. (**D**) Differential genes of carotenoid biosynthesis. (**E**) Differential genes of flavonoid biosynthesis. (**F**) Differential genes of flavone and flavonol biosynthesis. Blue boxes represent down-regulated genes, red boxes represent up-regulated genes, green boxes represent plant-specific genes, and dark green boxes represent both up-regulated genes and down-regulated genes.

Further analysis showed that the expression levels of forty-five genes related to the photosynthesis pathway, twenty-five genes related to carbon fixation, ten genes related to the carotenoid biosynthesis, and ten genes related to the flavonoid biosynthesis, flavone, and flavonol biosynthesis pathway were down-regulated (Figure 7A–F, Supplementary Tables S3–S7). Moreover, the RNA-seq results indicate that most of the genes associated with the tricarboxylic acid cycle (TCA cycle), galactose metabolism, fatty acid degradation, and valine, leucine, and isoleucine degradation were up-regulated in *osrne-1* (Supplementary Figure S4). These results indicate that loss-of-function mutation of OsRNE mainly affects the expression of photosynthesis, carbohydrate metabolism, and pigment-related genes.

### 2.7. OsRNE Mutation Affects the Expression of the Genes Involved in Chlorophyll Metabolism and Chloroplast Development

**Figure 8:**
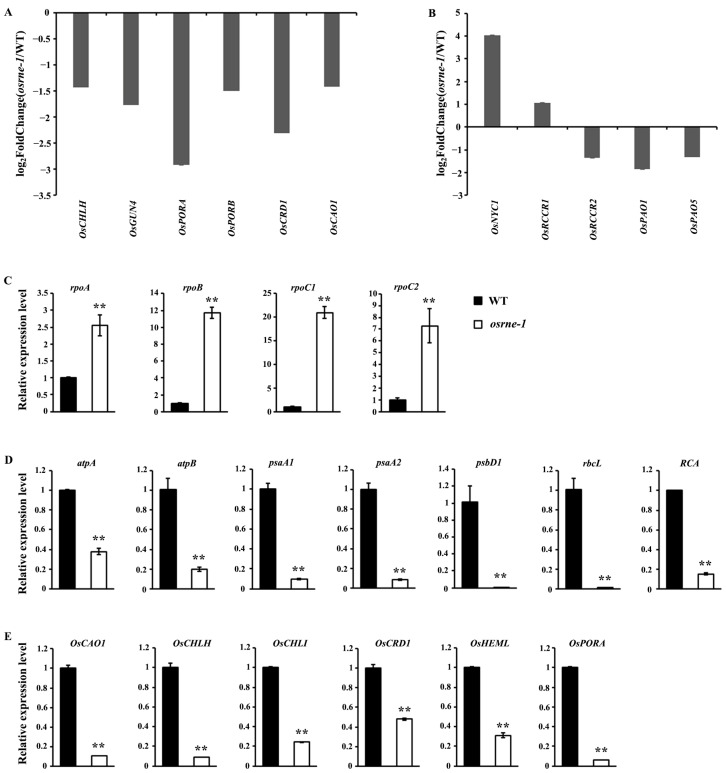
Differential expression of chlorophyll metabolism and chloroplast development genes in WT and *osrne-1*. The graphs show the log_2_ ratio of transcript levels involved in chlorophyll biosynthesis (**A**) and chlorophyll degradation pathway (**B**) in *osrne-1* compared with WT. Raw data are shown in Supplementary Tables S8 and S9. (**C**) Expression level of genes related to chloroplast biogenesis using qRT-PCR. (**D**) Expression level of genes related to photosynthesis using qRT-PCR. (**E**) Expression level of genes related to chlorophyll synthesis using qRT-PCR. The relative expression level of each gene was normalized using *Actin* as the internal control. Error bars are based on three independent biological replicates. Asterisks indicate statistically significant differences compared to the WT (** *p* < 0.01).

Due to *OsRNE* mutation leading to white leaf phenotype, the expression levels of chlorophyll metabolism genes in *osrne*-1 and WT were compared from the transcriptome data. The expression of many chlorophyll metabolism genes showed significant differences between *osrne*-1 and WT (Figure 8A,B, Supplementary Tables S8 and S9). Common pathway genes of chlorophyll synthesis, including *OsCHLH* (*chlorophyllide a oxygenase H subunit*), *OsGUN4* (*genomes uncoupled 4*), *OsPORA* (*protochlorophyllide oxidoreductase A*), *OsPORB* (*protochlorophyllide oxidoreductase B*), *OsCRD1* (*Oryza sativa dicarboxylate diiron protein 1*), and *OsCAO1* (*chlorophyllide-a oxygenase 1*), were strongly decreased in *osrne-1* (Figure 8A). In the chlorophyll degradation pathway, *OsNYC1* (*non-yellow coloring 1*) and *OsRCCR1* (*red chlorophyll catabolite reductase 1*) showed an up-regulated transcript level in *osrne-1*, while the expression levels of *OsRCCR2* (*red chlorophyll catabolite reductase 2*), *OsPAO1* (*polyamine oxidase 1*), and *OsPAO5* (*polyamine oxidase 5*) were significantly decreased in *osrne-1* (Figure 8B). To further study the effects of the *OsRNE* mutation on the expression of other genes associated with chlorophyll biosynthesis and chloroplast development, four chloroplast development genes (*rpoA*, *rpoB*, *rpoC1*, and *rpoC2* (four subunits of the plastid-encoded polymerase, PEP)), seven photosynthetic-related genes (*atpA*, *atpB*, *psaA1*, *psaA2*, *psbD1* (three reaction-center polypeptides), *rbcL*, and *RCA* (*Rubisco activase*)), and six chlorophyll biosynthesis genes (*OsCAO1*, *OsCHLH*, *OsCHLI*, *OsCRD1*, *OsHEML* (*glutamyl tRNA reductase*), and *OsPORA*) were selected for qRT-PCR assay (Figure 8C–E). The transcript abundance of *rpoA*, *rpoB*, *rpoC1*, and *rpoC2* involved in chloroplast development significantly increased in *osrne-1* (Figure 8C), while the expression of photosynthetic-related genes, including nuclear-encoding gene (*RCA*) and plastid-encoding genes (*atpA*, *atpB*, *psaA1*, *psaA2*, *psbD1*, and *rbcL*), significantly decreased in *osrne-1* (Figure 8D). In addition, the transcription level of chlorophyll biosynthesis genes (*OsCAO1*, *OsCHLH*, *OsCHLI*, *OsCRD1*, *OsHEML*, and *OsPORA*) decreased significantly in *osrne-1* (Figure 8E).

## 3. Discussion

Normal chloroplast development and chlorophyll biosynthesis are vital for the growth and development of green plants. RNase E is a key regulator of post-transcriptional gene expression, facilitating RNA degradation and the maturation of rRNAs and tRNAs in prokaryotes [16]. In *Arabidopsis*, loss of RNase E results in pale-green mutants with reduced plastid size, fewer thylakoids, and shorter granal stacks [28]. Processing of chloroplast transcripts still occurs in the *Arabidopsis* mutants, but some unprocessed high-molecular-weight transcripts accumulate [20]. Unlike in bacteria, *Arabidopsis* RNase E is not essential for survival [20,32]. Loss of RNE in *Arabidopsis* is perhaps partially compensated either by endonuclease RNase J or by other RNases rendering the mutant viable. As in the nuclear genome of *Arabidopsis*, there is only one RNase E/G gene in the nuclear genome of rice and not in the eukaryotes lacking chloroplasts [19,28]. Therefore, it is intriguing to determine the role of RNase E in rice. In this study, we proved that OsRNE is required for chloroplast development in rice by creating the *RNase E* mutants *wll1* and *osrne* in rice, which both display a lethal, chlorophyll-deficient, and white seedling phenotype (Figure 1 and Figure 2). The *osrne* mutant harbors a single base insertion (an A or T) that causes the frame shifts with premature termination of OsRNE (Figure 2). This might disable the enzymatic activity of OsRNE protein and lead to a defect in RNA processing in the chloroplasts. Interestingly, *Arabidopsis* RNase E mutant plants require sucrose at all stages of growth and flowering in vitro, allowing for seed harvest [19,28]. However, the rice *osrne* mutant still displayed an albino phenotype on MS medium containing 2% sucrose and failed to survive beyond the three-leaf stage, possibly because *Arabidopsis atrne* mutants on sucrose-containing media develop pale-green leaves and can synthesize some pigments, whereas rice mutants with more severe chloroplast phenotypes remain albino and are unable to synthesize pigments. In future work, metabolomic analysis will be employed to screen for metabolic pathways that are particularly affected during chloroplast development.

Temporal and spatial expression of genes is in accordance to their functions. Our expression analysis showed that the transcript of *OsRNE* was abundantly found in green leaves in rice (Figure 5B), indicating that *OsRNE* has a predominant expression in photosynthetic organs. The *OsRNE* expression pattern suggests its important function in chloroplast development. TEM analysis of the chloroplast ultrastructure revealed that the *osrne* mutant had developmentally defective chloroplasts with a small amount of thylakoids or no thylakoids (Figure 4C–F). Thus, OsRNE may be involved in chloroplast biogenesis and participate in the formation of thylakoids during chloroplast build-up.

Transcriptome analysis using *osrne-1* and WT seedlings in rice showed that DEGs related to photosynthesis, thylakoid, carbon fixation, carotenoid biosynthesis, and flavonoid biosynthesis were enriched in GO and KEGG analysis (Figure 6C–F). These enrichment patterns of DEGs are consistent with the chlorophyll and photosynthesis deficiency in *osrne-1*. Moreover, the expression levels of both the genes for synthesis and degradation of valine, leucine, and isoleucine were increased (Figure 6E). Reports have shown that branched-chain amino acids are synthesized in chloroplasts [33,34,35,36]. We infer that the mutation in *OsRNE* may lead to defects in amino acid metabolism. The increased biosynthesis and degradation of valine, leucine, and isoleucine could maintain the valine, leucine, and isoleucine flow at a normal level for plant growth.

Chloroplast genes are transcribed by NEP and PEP [12,13]. Ordination of two-way transcription is essential for chloroplast development. The status of the chloroplast could influence the transcription of nuclear genes through retrograde signaling. In *osrne-1* plants, expression of many nuclear genes changed (Supplementary Tables S1 and S2). The RNA-seq results show that the expression of the most photosynthetic genes was reduced (Supplementary Table S3). Conversely, the expression of nuclear-encoded chloroplast development genes (*rpoA*, *rpoB*, *rpoC1*, and *rpoC2*) was increased (Figure 8C), indicating a feedback regulatory mechanism for chloroplast biogenesis-related genes. Some mRNA transcripts require ribosomal translation for efficient stabilization [37,38,39]. The reduced transcripts of *atpA*, *atpB*, *psaA1*, *psaA2*, *psbD1*, and *rbcL* might result from the inefficient PEP transcription activity (Figure 8D). Furthermore, the reduced transcript of *rbcL* and the reduction in RbcL protein level suggested that the *osrne* mutant was most likely defective in biogenesis of plastid ribosomes (Figure 3B and Figure 8D). Moreover, the decreased expression of genes in the chlorophyll biosynthesis pathway (*OsCAO1*, *OsCHLH*, *OsCHLI*, *OsCRD1*, *OsHEML*, and *OsPORA*) and photosynthesis (*atpA*, *atpB*, *psaA1*, *psaA2*, *psbD1*, *rbcL*, and *RCA*) was likely due to the disruption of chloroplast development. Consequently, chlorophyll *a* and *b* accumulation decreased (Figure 3A). Therefore, the white leaf phenotype may be attributable to a defect in chloroplast development, resulting in the inability to carry out photosynthesis.

In *Escherichia coli*, RNase E forms a multiprotein complex called degradosome with PNPase, RNA-helicase, and enolase, involved in the degradation of bacterial mRNAs [16]. *Arabidopsis* RNase E forms a high-molecular-weight complex and interacts with an RNA-binding protein RHON1, which supports RNase E function in the chloroplast [32,40]. Therefore, in future work, we will use protein–protein interactions assays to screen the interacting proteins of OsRNE to gain a more comprehensive understanding of the regulatory network involved in chloroplast development.

In conclusion, we report herein that OsRNE is crucial for plastid gene expression, chlorophyll biosynthesis, and chloroplast development in rice.

## 4. Materials and Methods

### 4.1. Plant Materials and Growth Conditions

The *wll1* mutant with white leaf phenotype was initially identified from a ^60^Co-irradiated mutant pool of the *japonica* rice cultivar Yandao 8. The *osrne* mutants were generated using the CRISPR/Cas9 system. A target sequence located within the first exon of *OsRNE* was selected and cloned into CRISPR/Cas9 vector. The CRISPR/Cas9 vector was then transferred into the rice *japonica* variety Zhonghua 11 *calli* via *A. tumefaciens*-mediated transformation [41]. Among the plants of the T_0_ generation, the various types of *OsRNE* were confirmed by Sanger sequencing. As the *wll1* and homozygous *osrne* mutation was seedling-lethal, heterozygous plants were grown for reproduction. All rice materials were planted in the experimental field of Yangzhou University in May and harvested in October under natural conditions.

### 4.2. MutMap+

MutMap+ was performed as described previously [29,42]. Briefly, 30 green seedlings and 30 white seedlings from the heterozygous plant population were selected for DNA extraction. The DNA from individual plants was equally mixed to construct two DNA pools: pool 1 and pool 2. The DNA samples of pool 1 and pool 2 were randomly broken into fragments with 350 bp for the library construction, and then, two libraries were re-sequenced using an Illumina HiSequation 2500 (Novegene, Tianjin, China) to product the short sequence reads as raw data. After quality control of raw data and genome mapping, SNP/Indel were detected and annotated by GATK3.8 [43].

### 4.3. Determination of Pigment Content

Equal weights of fresh leaves of WT and *osrne-1* plants from two-leaf stage seedlings were soaked in 95% ethanol for 48 h in darkness and used to determine chlorophyll contents according to a described method [44]. Supernatant absorbances at 665, 649, and 470 nm were measured using a Hitachi U-1800 Spectrophotometer (Tokyo, Japan). Three independent biological repeats were measured for each sample.

### 4.4. Transmission Electron Microscopy (TEM) Analysis

Transverse sections of WT and *osrne-1* leaves from two-leaf stage seedlings were fixed in 2.5% glutaraldehyde in a phosphate buffer and further fixed in 1% OsO_4_ three hours at 4 °C. Samples were further dehydrated through a series of ethanol (20%, 40%, 60%, 70%, 80%, 90%, 95%, and 100% ethanol, 10 min for each concentration) and then embedded in Spurr’s medium. Ultrathin sections (approximately 70 nm) were produced using Leica EM UC7 ultramicrotome (Wetzlar, Germany). Sections were double-stained with uranyl acetate and lead citrate for 20 min and viewed with a Hitachi H-7650 transmission electron microscope (Hitachi High-Technologies Corp, Tokyo, Japan).

### 4.5. RNA Isolation and qRT-PCR Analysis

The RNA for analyzing the expression pattern of *OsRNE* was sampled from WT roots, culms, leaves, leaf sheaths, and panicles at the heading stage, respectively, through an RNAprep pure plant kit (Tiangen, Beijing, China). For studying the effects of the *OsRNE* mutation on the expression of genes associated with chlorophyll metabolism and chloroplast development, total RNA was extracted from two-leaf stage seedlings of WT and *osrne-1*. Then, 2 µg of total RNA was reverse-transcribed by NovoScript^®^Plus All-in-one 1st Strand cDNA Synthesis SuperMix (gDNA Purge) (Novoprotein, Suzhou, China). qRT-PCR was performed according to a previous description [45]. The primer sequences of *OsRNE*, internal control gene *Actin*, chlorophyll metabolism, and chloroplast development genes are listed in Supplementary Table S10.

### 4.6. Subcellular Localization of OsRNE

The OsRNE^1−206^ coding sequence was cloned into pCAMBIA1305-GFP and pAN580-GFP to generate recombinant plasmid p35S:OsRNE^1−206^-GFP and pAN580:OsRNE^1−206^-GFP, respectively. The primer sequences used for OsRNE subcellular localization are shown in Supplementary Table S11. The recombinant plasmids p35S:OsRNE^1−206^-GFP and pAN580:OsRNE^1−206^-GFP were expressed in *Nicotiana benthamiana* leaves and rice protoplasts, respectively, as described previously [46]. The GFP signals were observed using a Zeiss LSM880 confocal laser microscope (Carl Zeiss, Oberkochen, Germany).

### 4.7. Protein Extraction and SDS-PAGE

WT and *osrne-1* leaves (100 mg) from two-leaf stage seedlings were separately ground into fine powder with liquid nitrogen, and the total proteins were extracted in 500 μL of extraction buffer (10% SDS, 1 M Tris-HCl, pH 8.0). SDS-PAGE detection was performed using the standard procedures. The proteins of WT and *osrne-1* were resolved in 10% SDS–polyacrylamide gel electrophoresis (PAGE) gels and stained with Coomassie Brilliant Blue R250 to examine the protein bands. To better present the results, 10 μL and 5 μL of total proteins from WT and *osrne-1* were loaded individually.

### 4.8. RNA-Seq Analysis

Total RNA from two-leaf stage seedlings of WT and *osrne-1* was separately extracted using the TRIzol reagent (Invitrogen, CA, USA) according to the manufacturer’s protocol. Three biological replicates were used. The purity and quantification of RNA were evaluated using NanoDrop2000 (Thermo Scientific, Waltham, MA, USA). The integrity of RNA was assessed using Agilent2100 Bioanalyzer (Agilent Technologies, Santa Clara, CA, USA). The transcriptome sequencing and analysis were conducted by OE Biotech Co., Ltd. (Shanghai, China). Q value < 0.05 and |log_2_(FoldChange)| ≥ 1 were set as the threshold for significantly differential expression genes (DEGs). The GO and KEGG pathway analysis were performed using R (v3.2.0), respectively.

## Data Availability

The raw transcriptome sequence data have been submitted to the NCBI Short Read Archive (SRA) with accession number <PRJNA1103341>.

## Supplementary Materials

The following supporting information can be downloaded at https://www.mdpi.com/article/10.3390/ijms26052375/s1.
