# Supplementary material for: OsRNE Encodes an RNase E/G-Type Endoribonuclease Required for Chloroplast Development and Seedling Growth in Rice
Source: Int J Mol Sci. 2025 Mar 6;26(5):2375. doi: 10.3390/ijms26052375 (PMC11900968; doi:10.3390/ijms26052375)
Supplement: Supplementary file 1 [file ijms-26-02375-s001.zip › Supplementary Figures.pdf]

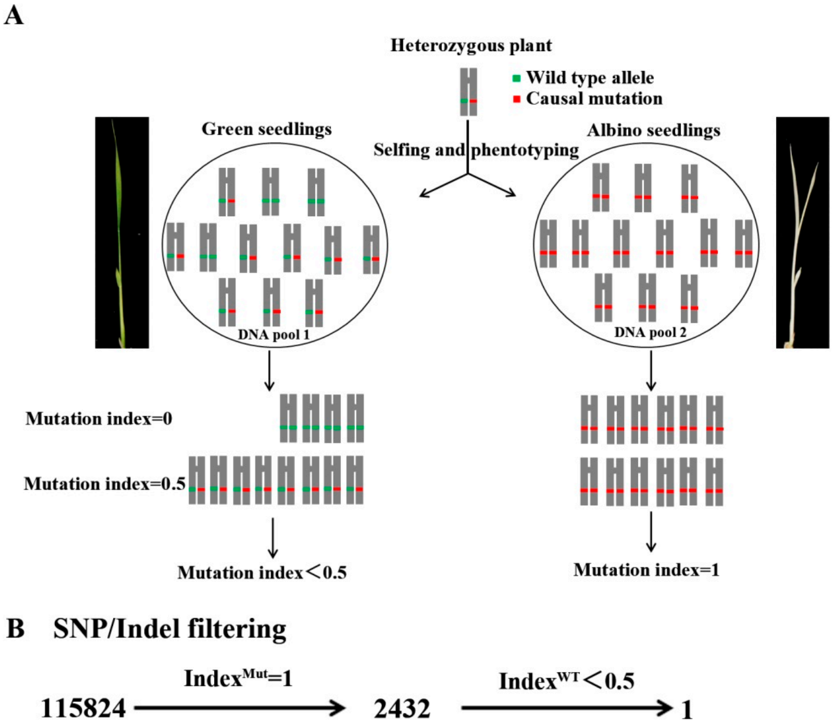

Supplementary Figure S1. Isolation of candidate mutations through MutMap+ and mutation filtering process. (A) Mutation filtering criteria for MutMap+. Grey bars symbolize homologous chromosomes. The red and yellow labels represent wild type allele and causal mutation, respectively. (B) The mutation filtering process of *wll1*. Filter parameters are indicated above the arrows. Numbers represent the quantity of SNP/Indel after each filtering.

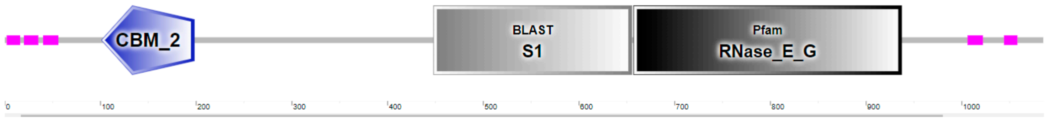

Supplementary Figure S2. Protein structure of OsRNE protein. Through http://smart.embl-heidelberg.de/, we found the deduced protein of OsRNE contained a carbohydrate-binding module 2 (CBM2) domain (101-198 aa) at the N terminus and an RNase_E_G motif (657-937 aa) at the C terminus. The pink rectangles represent low complexity sequences.

queryProtein WoLFPSORT prediction chlo: 11, mito: 2, pero: 1

PSORT features and traditional PSORTII prediction

14 Nearest Neighbors

id	site	distance	identity	comments
At5g04870.1	pero	206.677	14.0092%	[Arath]
At3g53920.1	chlo	208.901	11.7051%	[Arath]
PUR3_ARATH	chlo	243.415	9.21659%	[Uniprot] SWISS-PROT45:Chloroplast.
GPD2_ARATH	chlo	257.649	13.1797%	[Uniprot] SWISS-PROT45:Chloroplast.
SQD1_ARATH	chlo	261.222	11.9816%	[Arath] [Uniprot] SWISS-PROT45:Chloroplast. Evidence:IDA Pubmed:9465123
SECA_SPIOL	chlo	264.338	12.2711%	[Uniprot] SWISS-PROT45:Chloroplast stroma. A minor fraction is associated with the thylakoid membrane.
GCST_FLATR	mito	268.409	10.8756%	[Uniprot] SWISS-PROT45:Mitochondrial.
PHSL_VICFA	chlo	270.127	13.6823%	[Uniprot] SWISS-PROT45:Chloroplast; amyloplast.
GCST_SOLTU	mito	274.301	11.8894%	[Uniprot] SWISS-PROT45:Mitochondrial.
PURA_MAIZE	chlo	281.001	12.2581%	[Uniprot] SWISS-PROT45:Chloroplast.
At2g45300.1	chlo	281.502	13.7327%	[Arath]
LX21_HORVU	chlo	284.332	14.0884%	[Uniprot] SWISS-PROT45:Chloroplast.
AROA_ARATH	chlo	285.789	13.7327%	[Uniprot] SWISS-PROT45:Chloroplast.
LX23_HORVU	chlo	286.045	14.0092%	[Uniprot] SWISS-PROT45:Chloroplast.

Supplementary Figure S3. Subcellular Localization prediction of OsRNE in WoLF PSORT. Through https://wolfpsort.hgc.jp/, we found the protein of OsRNE most likely localized in chloroplast.

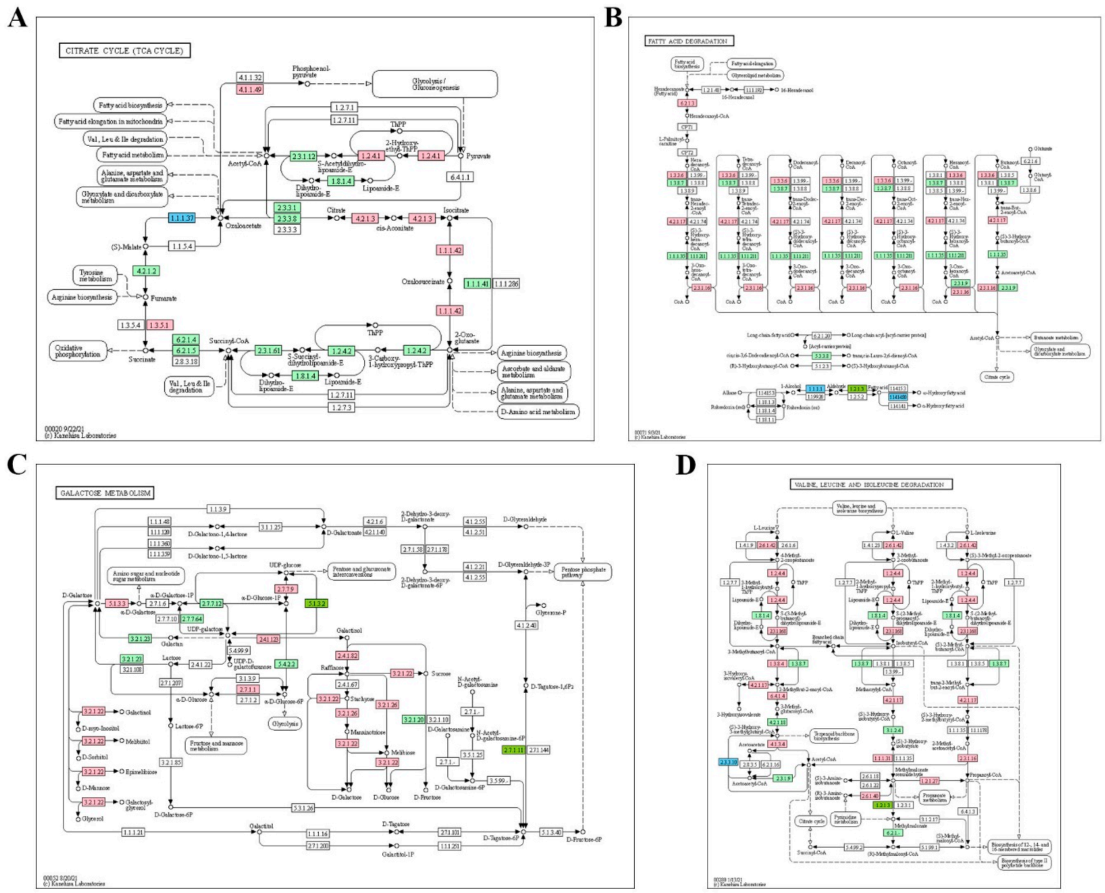

Supplementary Figure S4. Differential map of genes in citrate cycle (TCA cycle), galactose metabolism, fatty acid degradation, and valine, leucine and isoleucine degradation between wild type and *osrne* mutant. (A) Differential genes in citrate cycle (TCA cycle). (B) Differential genes in galactose metabolism. (C) Differential genes in fatty acid degradation. (D) Differential genes in valine, leucine and isoleucine degradation. Blue boxes represent down-regulated genes, red boxes represent up-regulated genes, green boxes represent plant-specific genes, and dark green boxes represent both up-regulated genes and down-regulated genes.
